# Lesion network mapping of focal injury-related aggression finds two distinct network injury patterns

**DOI:** 10.1093/braincomms/fcag032

**Published:** 2026-02-02

**Authors:** Gillian N Miller, Zexia Lu, Shaoling Peng, Isaiah Kletenik, Jorge Ortega-Márquez, Shreya Tripathy, Juliana Wall, Richard Ryan Darby, Alexander L Cohen

**Affiliations:** Department of Neurology, Boston Children’s Hospital, Harvard Medical School, Boston, MA 02115, USA; Department of Neurology, Boston Children’s Hospital, Harvard Medical School, Boston, MA 02115, USA; Department of Neurology, Boston Children’s Hospital, Harvard Medical School, Boston, MA 02115, USA; Division of Cognitive and Behavioral Neurology, Brigham and Women’s Hospital, Boston, MA 02115, USA; Department of Neurology, Brigham and Women’s Hospital, Boston, MA 02115, USA; Center for Brain Circuit Therapeutics, Brigham and Women’s Hospital, Boston, MA 02115, USA; Department of Neurology, Harvard Medical School, Boston, MA 02115, USA; Department of Neurology, Boston Children’s Hospital, Harvard Medical School, Boston, MA 02115, USA; Department of Neurology, Boston Children’s Hospital, Harvard Medical School, Boston, MA 02115, USA; Boston University Chobanian and Avedisian School of Medicine, Boston, MA 02118, USA; Developmental Neuropsychology Laboratory, Department of Psychology, University of Houston, Houston, TX 77204, USA; Department of Neurology, Vanderbilt University, Nashville, TN 37232, USA; Department of Neurology, Boston Children’s Hospital, Harvard Medical School, Boston, MA 02115, USA; Center for Brain Circuit Therapeutics, Brigham and Women’s Hospital, Boston, MA 02115, USA; Department of Neurology, Harvard Medical School, Boston, MA 02115, USA

**Keywords:** aggressive behaviour, crime, irritability, apathy, inhibition

## Abstract

Aggressive behaviour is prevalent across neurodevelopmental and neuropsychiatric conditions and is related to poor outcomes. Yet, despite extensive neuroimaging studies, a consistent set of brain networks where dysfunction is consistently related to aggressive behaviour remains unclear. Many studies are correlational in nature, while causal studies, such a lesion-location-based studies, are often limited to injury of the frontal lobes. Here, we analyse 61 focal brain lesions, identified in the current medical literature, that are associated with new-onset aggression and related behaviours. Lesions were traced onto a standardized brain atlas and used as seeds for a functional connectivity analysis, leveraging resting-state data from 1000 healthy individuals. These maps, representing likely pre-injury connectivity, were grouped using a data-driven hierarchical clustering approach. Then, the lesion networks of the identified clusters were separately compared with the connectivity of 716 lesions causing other symptoms. This data-driven approach identified two distinct lesion subgroups that both appear to manifest aggression through the co-occurrence of disrupted functional connectivity to networks involved in emotional expression and cognitive control. Both ‘aggression networks’ demonstrated sensitivity and specificity when compared with lesions causing a wide variety of other neuropsychiatric symptoms. The first subgroup network involved connectivity to the anterior cingulate cortex and was correlated with the connectivity of lesions causing akinetic mutism. The second subgroup network involved connectivity to the ventromedial prefrontal cortex and contained a notable subset of cases (*n* = 25) that had reported criminal behaviour, supporting a role of self-control in this subgroup and implying separable influences towards criminality within our identified aggression networks. Alterations within the first group aligned with motivation networks, while the second group aligned with disinhibition networks, indicating these as the potential underlying factors in aggression in the two subgroups, respectively. This characterization was supported by previous work on the atrophy network mapping of behavioural variant frontotemporal dementia. Overall, this study suggests that aggressive behaviour may be more likely after injury to distinct brain networks, which may be related to distinct behavioural factors, and has implications for potential targeted therapeutic interventions such as focused neuromodulation.

## Teaser

Miller *et al*. report that post-lesion aggression involves disruptions in two distinct brain networks identified through lesion network mapping: motivation-related anterior cingulate circuits and disinhibition-related ventromedial prefrontal networks. Supported by associations with behavioural variant frontotemporal dementia and other lesion-based studies, these findings suggest aggression arises from network-specific mechanistic disruptions.

## Graphical Abstract

**Graphical Abstract:**
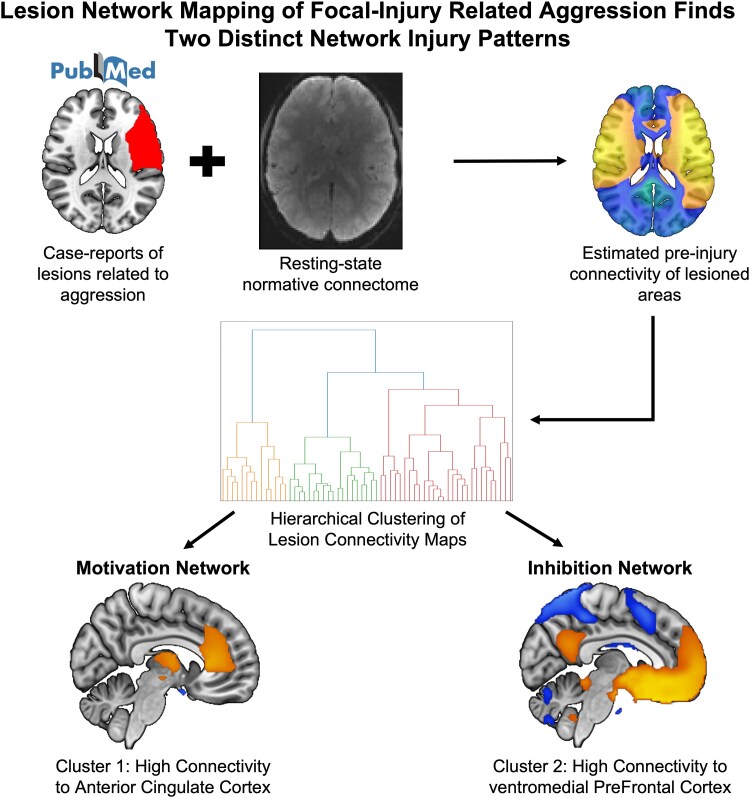

## Introduction

The term ‘aggression’ encompasses a range of behaviours leading to physical or verbal harm, or threat of harm, to oneself, others or objects.^1,2^ Aggressive behaviour has been related to poor social outcomes,^3,4^ reduced treatment response^5^ and can even predict future depressive symptoms.^6^ In addition, comorbid aggressive behaviours can have a significant impact on caregiver burden,^7,8^ as well as on nursing staff and quality of medical care.^9^ Aggressive behaviours are common in many neurodevelopmental conditions, including autism spectrum disorder, Down Syndrome, tuberous sclerosis complex and attention-deficit/hyperactivity disorder,^4,10,11^ and are occasionally the primary symptom of concern, as in disruptive mood dysregulation disorder.^12^ Similarly, aggression is also common in a number of psychiatric conditions such as depression, bipolar disorder and schizophrenia^13-15^ Given its prevalence and profound impact, understanding the neural substrates of aggressive behaviour may aid in early diagnosis and stratification. Of note, the development of new targeted treatment strategies may become possible as well—preliminary results in healthy adults have shown promise in using non-invasive brain stimulation to modulate aggressive behaviour.^16^

Neuroimaging studies employing various methods—including task and resting-state function MRI (fMRI), structural connectivity, meta-analysis and voxel-wise lesion symptom mapping—across a wide range of populations, ranging from healthy controls to patients in psychiatric or correctional facilities, have variably implicated multiple brain regions in aggression. Functional and structural connectivity-based neuroimaging studies have identified differences across the brain, in all four lobes as well as in subcortical structures (e.g. thalamus and amygdala).^17-21^ Unfortunately, most findings from traditional cross-sectional neuroimaging studies are correlational, making it difficult to determine whether an observed brain difference precedes and contributes to aggression (i.e. is causal) or instead emerges in response to it—either as a compensatory adaptation or simply as an accompanying, non-causal change. Conversely, there are studies of aggression following brain injury that provide a stronger causal inference, as the emergence of new symptoms can be temporally linked to the site of neural disruption,^22^ though these studies are often limited in aetiology and affected regions. Lesion-location-based studies have largely focused on aggression following traumatic brain injury^23-27^ and the involvement of frontal regions, such as the ventromedial frontal lobe and prefrontal cortex.^28,29^ However, this finding, while sensitive, may be somewhat non-specific, as studies have linked aggression to regions throughout the frontal lobe, including the dorsolateral prefrontal cortex (dlPFC), ventromedial prefrontal cortex (vmPFC), orbitofrontal cortex (OFC) and medial frontal cortex.^17^ Yet not all patients with frontal lobe injury develop aggression^30^ and non-invasive brain stimulation of frontal regions has not been overwhelmingly successful in moderating aggression.^16^ Given this variability, it is possible that the development of aggression is a network phenomenon rather than the result of dysfunction in one specific location. Indeed, studies have found that lesions outside of the frontal lobe, such as in regions of the temporal lobe, are also related to aggression, aligning with fMRI findings.^17^ Recently, we were able to integrate fMRI-based network findings with lesion-based findings using a dataset of penetrating head injuries.^31^ However, it remained unclear whether similar network-level alterations are present across lesions of diverse aetiologies. Given the complexity and individual variability of the neural systems involved in aggressive behaviour, identifying which network alterations are most consistently implicated is the purpose of the current investigation.

To determine whether similar network-level patterns emerge across heterogeneous lesion aetiologies, we apply lesion network mapping, a network-based method that leverages data to investigate lesions associated with new-onset aggression. Lesion network mapping identifies whether specific brain networks are associated with a particular symptom or behavioural outcome by combining a large cohort of lesions associated with said symptom and a high-quality map of typical brain network organization, i.e. a functional ‘connectome’.^32^ Here, we use lesion network mapping to derive one or multiple ‘aggression network(s)’ using all available case reports and case series of lesion-associated aggressive behaviour in the medical literature. In addition, given the highly heterogeneous nature of aggression and related behaviours, we determine whether there are distinct relationships between lesion connectivity and described aggression phenotypes.

## Materials and methods

### Patient cases from the literature

We sought to identify all reported cases in the current literature of patients with new-onset aggression or related behaviours after a focal brain lesion where an imaging depiction of lesion location was included. Potential cases were assessed based on the following inclusion criteria: (i) a case description of post-injury aggression, anger, agitation and/or irritability in a patient; (ii) a focal brain lesion; and (iii) a published image of the lesion of sufficient quality to allow tracing of the lesion’s location onto a standardized brain atlas.

We performed a systematic review via a PubMed search using the terms (brain) AND (case report) AND (aggression) AND (MRI OR CT OR neuroimaging) AND (lesion OR lesions OR stroke OR infarct* OR ischem* OR ischaem* OR hemorrhag* OR haemorrhag* OR tumor OR tumour). This identified 1354 potential papers for inclusion. Each abstract was reviewed to determine case relevance; based on this, 37 records were selected for full text review. Of these, 24 papers representing 26 cases met the inclusion criteria.

Following this, a secondary PubMed search was conducted using the terms (brain) AND (aggression OR irritative OR hostile OR violence OR offensive OR angry) AND (MRI OR CT OR neuroimaging) AND (lesion OR stroke OR infarct* OR ischem* OR hemorrhag* OR tumor). This second search yielded 3837 papers, with many duplicates from the original search. These abstracts were reviewed as above, providing 20 additional cases from 17 reports. Finally, an additional 15 patients from 4 reports were obtained from references in the above included papers. Of these, 11 patients did not have behavioural descriptions available in the original report but were included in the current study due to the violent nature of their reported crimes (e.g. murder, aggravated assault, manslaughter). Overall, this resulted in 61 patients for inclusion in our analyses (Supplementary Table S1).

Lesions were traced by hand onto the MNI152 sixth generation atlas (1 mm resolution) available in FSL v6.0^33^ using 3DSlicer v5^34^ to create ‘2D lesion masks’ for all subjects in the same plane as each figure.^35^ In cases where the lesion was shown in more than one 2D slice, all slices were traced and combined into a single mask (Supplementary Fig. S1). G.N.M., Z.L., S.P. and J.W. completed primary lesion tracings which were reviewed by A.L.C., a trained neurologist with neuroimaging expertise.

### Lesion network mapping

#### Lesion connectivity maps

These 61 lesion masks were then used as individual seeds in a resting-state functional connectivity analysis using fMRI data collected from the Genome Superstruct Project (GSP) dataset of healthy young adults.^36,37^ Since fMRI data are not available from patients ‘prior’ to their brain injury, a normative (population-average) functional connectivity map was created for each lesion by correlating the average pre-processed time course of the lesion location with the time course of every other brain voxel in each of 1000 healthy young adult participants.^38,39^ Then, the average correlation and statistical *t*-score map (T-map) for each lesion mask was calculated across these 1000 functional connectivity maps—creating a map of likely/typical pre-injury connections to each lesion location.

#### Lesion network overlap

To assess the consistency of strong connections within this sample, a ‘lesion network overlap’ map was created by thresholding the 61 lesion T-maps at T > ±9 [equivalent to a voxel-wise family-wise error (FWE) corrected *P*-value <10−^11^] to create a binarized map of functionally connected regions to each patient’s lesion site.^37^ These binarized maps were then overlapped to find the number of patients with lesions functionally connected to each voxel at the selected threshold.

### Lesion network specificity

We investigated the specificity of our ‘aggression network’, i.e. the consistently detected connections of our cohort, by comparing lesion connectivity of our cohort to that of a control dataset comprised of 716 lesions associated with 22 previously investigated neuropsychiatric syndromes from the Boston Lesion Repository (Supplementary Table 2). Two-sample *t*-tests were performed with the unthresholded lesion connectivity maps via non-parametric permutation testing (FSL PALM v. alpha109) using 2000 permutations, tail approximation^40^ and FWE corrected *P*-value <0.05. Each neuropsychiatric syndrome was modelled as an independent group, ensuring an unbiased comparison across different conditions despite varying sample sizes, with equal weights assigned to each group (i.e. leveraging a random-effect versus fixed-effect model).

### Sex-related network comparison

To assess for sex-related network differences, we compared the lesion connectivity of our male patients to that of our female patients with a two-sample *t*-test using the unthresholded lesion connectivity maps via non-parametric permutation testing (FSL PALM v. alpha109) and 2000 permutations, tail approximation^33^ and FWE corrected *P*-value <0.05.

### Data-driven clustering

Since aggression encompasses a wide range of heterogeneous behaviours, we performed an exploratory data-driven clustering of the 61 lesion connectivity maps to identify whether there were distinct subgroups within the cohort. We first computed the pairwise spatial correlation between each patient’s functional connectivity T-map, then performed hierarchical clustering on the Euclidean distance matrix derived from the resulting correlation matrix.^41^ Based on the results of this clustering, we repeated the lesion network overlap as well as the comparison to lesions causing other symptoms as above for identified subgroups.

### Cluster characterization

Blinded to cluster-membership, two independent raters (G.N.M. and I.K.) examined case descriptions to identify keywords related to each patient’s behavioural presentation. Given the distinction between reactive and proactive aggression in the literature, special attention was paid to keywords that could help to classify cases on this axis. The results were then reviewed to determine whether distinct behavioural phenotypes emerged within identified subgroups.

Additionally, we compared the spatial correlation of each subgroup’s lesion network map to that of an aggression network identified independently using a different cohort of 182 patients with traumatic brain injury.^28,31^ Finally, we also calculated the spatial correlation of each cluster’s lesion network map with the lesion connectivity of the lesions related to the 22 other neuropsychiatric syndromes as previously mentioned. From this analysis, we identified the syndromes with the highest average correlation with each cluster. We then used two-tailed, paired *t*-tests to determine whether the correlation of lesion connectivity of these identified syndromes was significantly different between the clusters.

### Comparison to criminal behaviour

Of note, our patient cohort contained 29 patients who were also included in a previous investigation of patients with lesion-associated criminal behaviour.^42^ Given this overlap, we conducted additional analyses to determine whether there was separable and unique network involvement leading to crime versus aggression. Within the cluster that showed overall similarity to the criminal behaviour network, additional testing was performed wherein patients were classified based on case descriptions mentioning criminal activity. As above, we repeated lesion network overlap and lesion network specificity analyses for these two groups, as well as directly compared them to each other with a two-sample *t*-test of the unthresholded lesion connectivity maps via non-parametric permutation testing (FSL PALM v. alpha109) using 2000 permutations, tail approximation^40^ and false discovery rate (FDR) corrected *P*-value <0.005.

### Statistical analysis

We took multiple steps to ensure the reproducibility and generalizability of our results. We varied the thresholds of our lesion network overlap approach to confirm stability of our localization and used a non-parametric, permutation-based approach to perform statistical tests. Additionally, we used FWE rather than FDR correction where appropriate. Finally, we used a single software package for each type of analysis in accordance with existing best practice recommendations.^43^ We note that these steps reduce but do not fully eliminate the risk of false positives.^43,44^

## Results

### Lesions associated with aggression localize to a brain network that is both sensitive and specific

**Figure 1:**
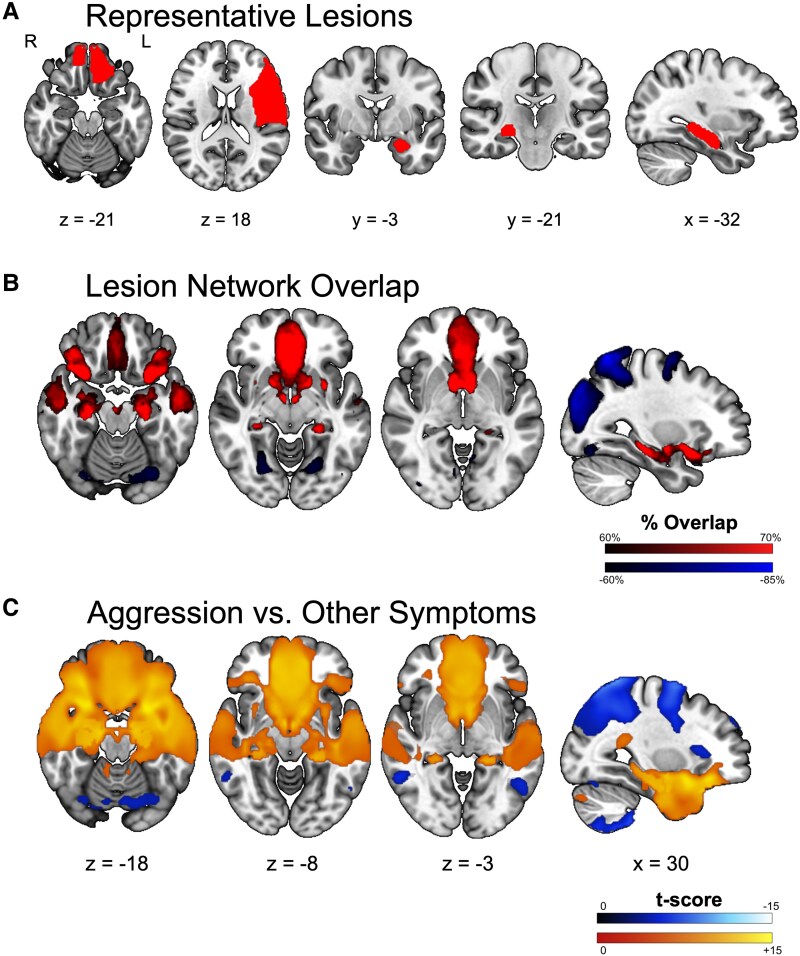
**Lesion network localization of aggression.** (**A**) Five representative lesions (of 61 total) causing aggression, demonstrating heterogeneity of lesion location. (**B**) Voxels functionally connected at least 60% of lesions (*n* = 61 lesions). (**C**) Two-sample *t*-test comparing functional connectivity lesions causing aggression (*n* = 61 lesions) and a large cohort of lesions causing other symptoms (*n* = 716 lesions) (FWE corrected *P* < 0.05).

A systematic literature review identified 61 cases of aggression or related behaviour suspected to be related to an identified brain lesion. The cases demonstrated a male predominance (M = 47, 77.1%), with lesions occurring across the lifespan (mean age = 33.04 years, SD = 19.56). The 61 lesions were spatially diverse, with no single brain region lesioned in even a simple majority of cases (Fig. 1A). The most commonly lesioned locations were the frontal lobe (*n* = 23) and temporal lobe (*n* = 22). Lesions showed no bias towards either hemisphere; 31.2% of lesions were right lateralized (*n* = 19), 27.9% were left lateralized (*n* = 17) and 41.0% were bilateral (*n* = 25).

The brain regions functionally connected to each lesion were computed and compared across the cohort. We found that 70% (*n* = 42) of lesions associated with aggression were positively correlated with the vmPFC (0, 34, −8), bilateral lateral OFC (± 30, −13, −16) and the bilateral posterior hippocampus (±26, −20, −16). Lesions associated with aggression were also negatively correlated with occipital and superior parietal regions (Fig. 1B). Further analysis demonstrated that these connections were statistically significant when compared with the 716 lesions related to 22 other specific symptoms (Fig. 1C and Supplementary Fig. S2). However, while statistically significant, the level of consistency or overlap of the network was lower compared with several prior lesion network mapping analyses,^42,45,46^ prompting additional investigation to understand the makeup of this cohort.

#### The identified brain network did not show significant sex-related differences

Of note, there were no voxels of significant difference when comparing the connectivity of lesions between male and female patients after correction for multiple comparisons. Regions of difference that were significant before correction were not in the key nodes (Supplementary Fig. S3).

### Lesions associated with aggression form two distinct clusters based on network connectivity

Based on the statistical significance but low level of consistency of the overall group, we explored whether coherent subgroups existed in our data. The original 61 lesions had a low average cross-correlation of *r* = 0.24 and average internal variance of 7.84. Hierarchical clustering of the lesions’ functional connectivity maps revealed two distinct groups with increased homogeneity. The first group included 14 lesions with an average cross-correlation value of *r* = 0.53 and an average internal variance of 3.14. Patients forming this cluster were 64.3% male (*n* = 9); lesions were 42.9% right lateralized (*n* = 6), 35.7% left lateralized (*n* = 5) and 21.4% bilateral (*n* = 3). The second identified group included 47 lesions with an average cross-correlation value of *r* = 0.35 and an average internal variance of 5.94. Patients forming this cluster were 80.9% male (*n* = 38); lesions were 27.7% right lateralized (*n* = 13), 25.5% left lateralized (*n* = 12) and 44.7% bilateral (*n* = 21).

**Figure 2:**
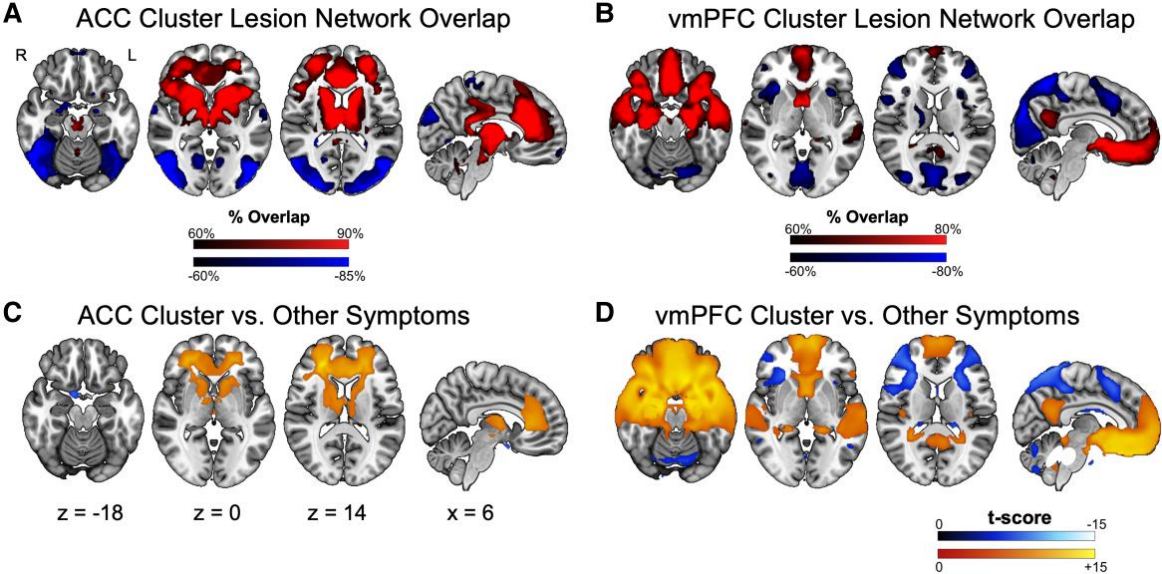
**Lesion network localization of each cluster**. (**A**) Voxels functionally connected at least 60% of lesions in the ACC Cluster (*n* = 14 lesions). (**B**) Voxels functionally connected at least 60% of lesions in the vmPFC Cluster (*n* = 47 lesions). (**C**) Two-sample *t*-test comparing functional connectivity of the ACC Cluster (*n* = 14 lesions) to the large cohort of lesions causing other symptoms (*n* = 716 lesions) (FWE corrected *P* < 0.05). (**D**) Two-sample *t*-test comparing functional connectivity of the vmPFC Cluster (*n* = 47 lesions) to the large cohort of lesions causing other symptoms (*n* = 716 lesions) (FWE corrected *P* < 0.05).

The functional connectivity patterns of the first group indicated that 90% of the lesions were positively correlated with the anterior cingulate cortex (ACC: 0, 28, 25), regions of the bilateral anterior insula (±43, 19, 2), regions of the bilateral dlPFC (±29, 48, 10), the bilateral anterior thalamus (±11, −9, −13) and regions of the basal ganglia, including the superior caudate (±12, 12, 8), anterior middle putamen (±23, 10, −2) and the anterior globus pallidus (±19, 3, −1). Lesions were negatively correlated to regions of the bilateral lateral occipital lobe (±50, −72, 2; Fig. 2A). Connections to the ACC, basal ganglia and dlPFC were statistically significant when compared with lesions causing other symptoms (Fig. 2C). We refer to this group as the ‘ACC Cluster’.

The functional connectivity patterns of the lesions in the second group indicated that 80% of the lesions were positively correlated with the (vmPFC: 0, 34, −8), bilateral lateral OFC (± 30, −13, −16), the bilateral anterior (±24, −8, −23) and posterior (±26, −20, −16) hippocampus, the bilateral posterior amygdala (±21, −6, −20), bilateral temporal pole (±40, 22, −32) and bilateral middle temporal gyrus (±53, −3, −19). Lesions were negatively correlated with the right supramarginal gyrus (54, −33, 53; Fig. 2B). Connections to these regions were statistically significant when compared with lesions causing other symptoms (Fig. 2D). We refer to this group as the ‘vmPFC Cluster’.

#### Behavioural characterization of clusters

Analysis of the case descriptions did not identify any specific behavioural patterns that differentiated the two groups, including irritability, lack of remorse/empathy/guilt, impulsivity, sexual or social dysfunction or mood lability. This could be due to the lack of detailed, systematic reporting across the cases and/or because the two networks largely converge on a shared overarching behavioural phenotype with more subtle and nuanced differences. One potential exception was criminal behaviour; 35.7% (*n* = 5) of patients in the ACC Cluster were recorded as having committed a crime at the time of report, compared with 53.1% (*n* = 25) of patients in the vmPFC Cluster. Here, as we are now aiming to characterize the overall behavioural profile of the patients, we include all crimes leading to arrest rather than just violent crimes. Non-violent crimes in this study cohort include petty theft, drug abuse and property destruction. Of note, a majority of the cohort (across both subgroups) seemed to present with reactive/impulsive rather than proactive aggression,^47^ when case descriptions were detailed enough for a judgement to be made.

#### Network-based characterization of clusters

**Figure 3:**
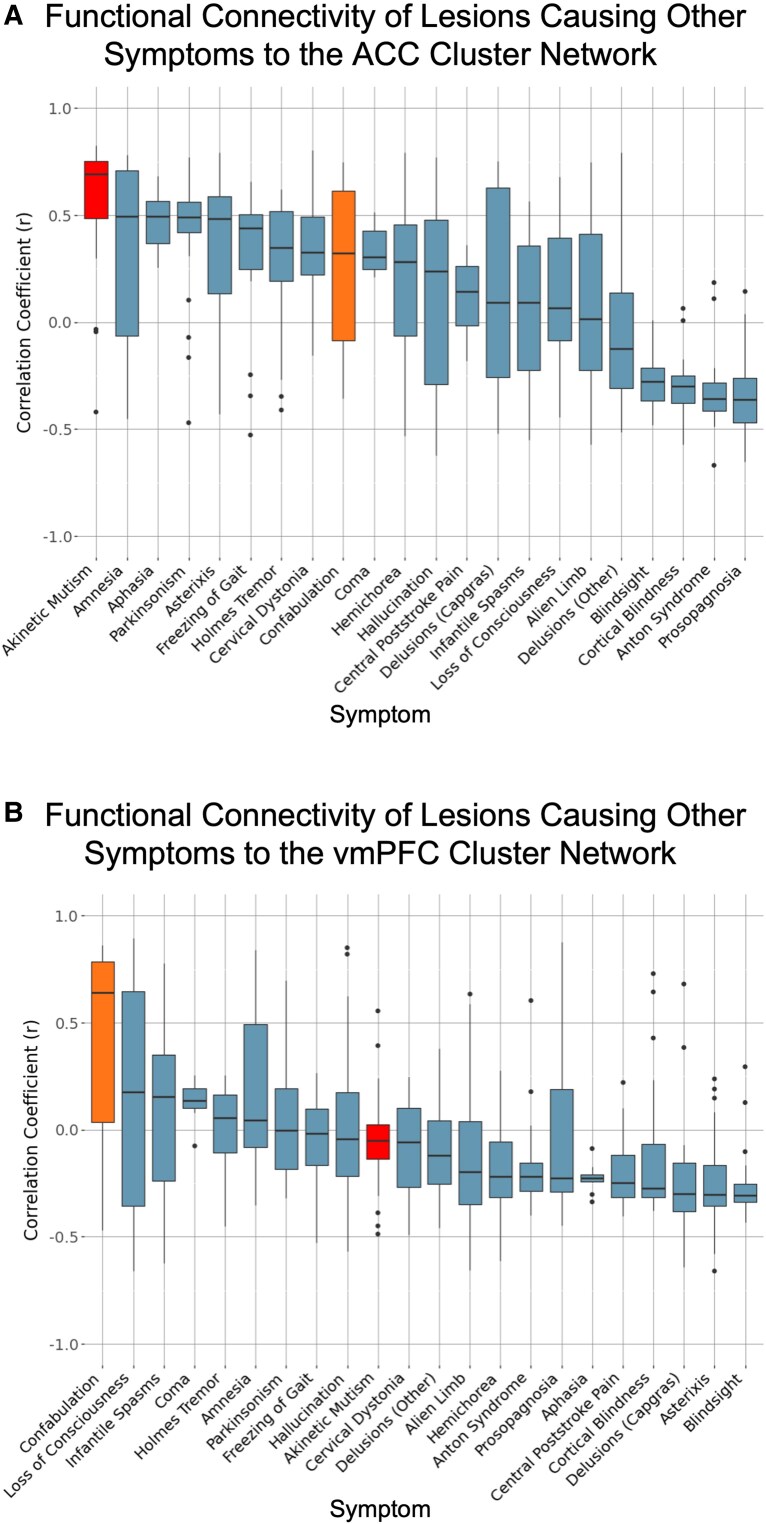
**Alignment of cluster networks with lesions causing other symptoms.** Box plots showing the distribution of correlation coefficients between connectivity maps derived from individual lesions (*n* = 716 total lesions) associated with 22 clinical syndromes and each cluster network. Each data point represents the correlation coefficient for a single lesion-derived connectivity map, and each box represents the distribution of lesion-level correlations within a syndrome. (**A**) Correlation of syndrome lesions and the ACC cluster network map. Akinetic mutism showed the strongest correlation (average *r* = 0.56) with this cluster network. Paired *t*-tests revealed that lesions causing akinetic mutism (shown in at far left in red) were significantly more correlated to the ACC Cluster network compared with the vmPFC Cluster network [*t*(27) = 11.678, *P* < 0.0001]. (**B**) Correlation of syndrome lesions and the vmPFC cluster network map. Confabulation showed the strongest correlation (average *r* = 0.46) with this cluster network. Paired *t*-tests revealed that lesions causing confabulation (shown in orange, ninth from the left) were not significantly more correlated to the vmPFC Cluster network compared with the ACC Cluster network [*t*(24)=−1.056, *P* = 0.302].

The ACC Cluster network was more correlated (*r* = 0.55) with an independently derived single-cohort aggression network recently published by our group^31^ than the vmPFC Cluster network (*r* = 0.16). When compared with connectivity patterns of lesions causing other symptoms, the ACC Cluster network was most correlated with lesions related to akinetic mutism (‘average’ *r* = 0.56)^46^ and the vmPFC Cluster network was most correlated with lesions related to confabulation (‘average’ *r* = 0.46; Fig. 3).^45^ Paired *t*-tests revealed that the correlations with lesions related to akinetic mutism were significantly different between the clusters [*t*(27) = 11.678, *P* < 0.0001], however the correlations with lesions related to confabulation were not [*t*(24)=−1.056, *P* = 0.302].

### Aggression and criminal behaviour map onto overlapping, but distinct networks

**Figure 4:**
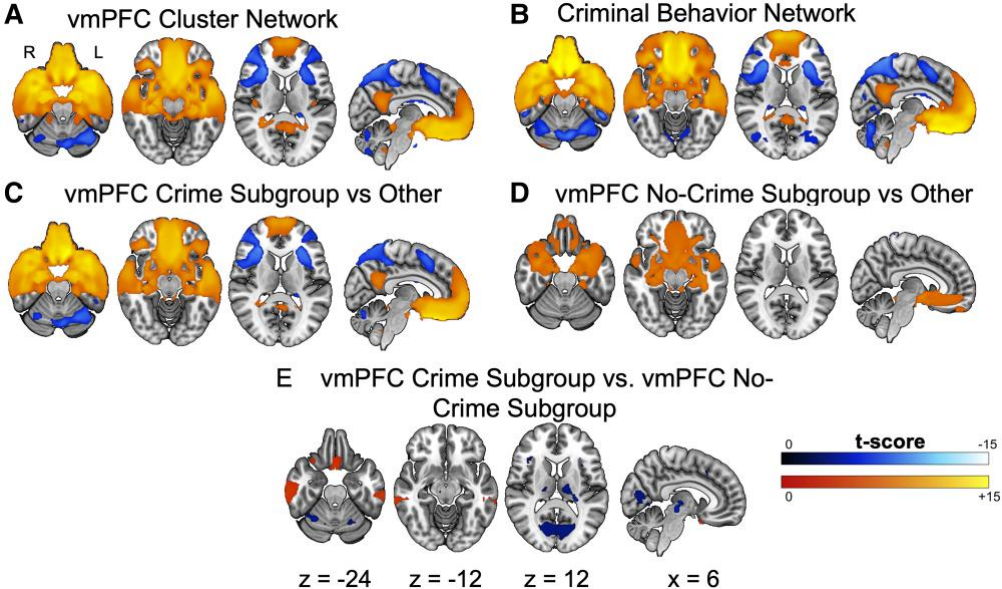
**Lesion network localization of criminal behaviour in the vmPFC cluster.** (**A**) Functional connectivity of vmPFC Cluster lesions (*n* = 47 lesions) compared with lesions causing other symptoms (*n* = 716 lesions) (FWE corrected *P* < 0.05). (**B**) Functional connectivity of 40 lesions related to criminal behaviour compared with lesions causing other symptoms (*n* = 716 lesions) (FWE corrected *P* < 0.05). (**C**) Two-sample *t*-tests comparing functional connectivity of crime subgroup (*n* = 25 lesions) to the large cohort of lesions causing other symptoms (*n* = 716 lesions) (FWE corrected *P* < 0.05). (**D**) Two-sample *t*-tests comparing functional connectivity of no-crime subgroup (*n* = 22 lesions) to the large cohort of lesions causing other symptoms (*n* = 716 lesions) (FWE corrected *P* < 0.05). (**E**) Two-sample *t*-test comparing functional connectivity of crime (*n* = 25 lesions) versus no-crime subgroups (*n* = 22 lesions) (FDR corrected *P* < 0.005).

The vmPFC Cluster contained 83.3% (*n* = 25) of the cases with reported criminal behaviour in our cohort (*n* = 30) and the resulting network map for this cluster (Fig. 4A) was notably similar to a previously published lesion network related to criminal behaviour (Fig. 4B).^42^ Therefore, we assessed unique contributions of crime versus aggression in our vmPFC Cluster by dividing the patients into those with reported criminal behaviour (*n* = 25) and those without (*n* = 22).

Two-sample *t*-tests against the control lesion dataset revealed that both subgroups shared specific positive connections to the vmPFC, OFC, hippocampus, amygdala and medial temporal pole (Fig. 4C and D). Direct comparison also revealed some areas of significant difference (FDR corrected *P* < 0.005) between the groups. The crime subgroup had stronger positive connections to regions of the inferior medial OFC and inferior temporal gyrus, and stronger negative connections to regions of the anterior insula, thalamus, basal ganglia (i.e. putamen, globus pallidus) and medial occipital lobes (Fig. 4E).

## Discussion

Many distinct regions in the brain have been associated or correlated with aggression in various paradigms and across multiple modes of investigation. Here, we used lesion network mapping to identify two distinct networks that are potentially associated with aggression, leveraging data from 61 patients with lesions believed to have caused aggression or related behaviours. One network was characterized by connectivity to the ACC, dlPFC and basal ganglia, while the second was characterized by connectivity with the ventral medial prefrontal cortex (vmPFC, OFC, temporal lobes and the hippocampus/amygdala. While distinct, the two cluster networks appear to converge on a shared behavioural phenotype: a model of aggression in which aggressive behaviour—specifically impulsive or reactive aggression—is generated by a combined disruption in emotional expression and cognitive control networks.^17^

While aggressive behaviour often shows sex-related differences,^48^ lesion connectivity patterns in our cohort did not significantly differ between males and females. However, our sample did show an overall male predominance. This male predominance may be due to a sex- or gender-related difference in the presentation of aggressive behaviour, which could in turn cause a bias in reporting. For example, previous work has found that females use more indirect aggression (i.e. ‘social manipulation with the intent to harm’), while males use more physical aggression.^49,50^ This difference may alter the clinical salience and description of patient behaviours. Notably, the vmPFC Cluster had 15% more males than the ACC Cluster. Since the vmPFC Cluster overlaps with criminal behaviour and males are often disproportionately represented in criminal convictions,^51^ this could indicate that sex-related interactions with the vmPFC network may amplify behavioural differences between males and females more compared with the ACC Cluster. Social roles,^52^ as well as genetics and hormones,^49^ appear to play a role in the presentation of aggressive behaviour and thus may also play a role in how injury to our identified ‘aggression networks’ manifests behaviourally.

Although aggression and related emotional states have traditionally been associated with left-hemispheric specialization, particularly within frontal regions,^30,53^ we did not observe a clear lesion lateralization effect in our cohort. Across the sample, lesions were most often bilateral, with right- and left-sided lesions occurring at similar rates, indicating no strong lateralization overall. This absence of hemispheric predominance suggests that lesion-related connectivity disruptions may interfere with broader network dynamics rather than affecting one hemisphere selectively, despite significant lateralization in the functional imaging literature. Aligned with this, prior work has shown that focal brain damage can induce widespread, non-local alterations to brain networks, including effects within the contralateral hemisphere.^54^ Interestingly, the ACC Cluster showed comparable numbers of left- and right-lateralized lesions, with fewer bilateral cases, whereas the vmPFC Cluster was characterized by a higher proportion of bilateral lesions and roughly equal numbers of left- and right-lateralized lesions. This difference may reflect differing vulnerability across the networks; for instance, greater disruption may be required within the vmPFC Cluster to observe clinically relevant behavioural effects, resulting in a higher prevalence of bilateral lesions reported in the literature. The lack of lateralization findings may also reflect methodological distinctions from prior activation-based imaging studies: lesion network mapping identifies regions whose ‘disruption’ is related to aggressive behaviour,^31^ whereas functional MRI detects regions that are more selectively activated during aggressive states.^55^ Consequently, lesion effects may highlight the importance of interhemispheric network integrity rather than focusing on the most specialized node of the network as in classical functional lateralization studies.

Our first cluster displays unique key connections with the ACC, dlPFC, anterior thalamus and regions of the basal ganglia (i.e. caudate, putamen and globus pallidus). The ACC is often considered a core region for executive control and many psychological processes with alterations in the ACC having been specifically linked to reactive/impulsive aggression.^56,57^ Similarly, the dlPFC has been characterized as a central control node of an emotion regulation network.^58^ Likewise, prior work indicates that the anterior thalamus contributes to emotional experience and expression, and the basal ganglia support the expression and downregulation of negative emotions, with alterations to the bilateral caudate, putamen and globus pallidus having also been specifically implicated in aggression and irritability^59-61^. Interestingly, both the ACC and basal ganglia function as relay centres in the brain, where information from ‘emotional’ and ‘cognitive’ regions is combined^62-65^. As such, aggression in this subgroup of patients may result from indirect alterations in emotional expression and cognitive control, caused by altered regulation of the regions directly involved.

The ACC Cluster network demonstrated the most functional network overlap with lesions causing akinetic mutism, suggesting that lesions associated with akinetic mutism and lesions associated with aggression via impact to the ACC Cluster network may disrupt some of the same networks—though likely through different mechanisms, as reflected in their distinct behavioural presentations. Akinetic mutism is an extreme form of abulia characterized by apathy and loss of spontaneous behaviour and speech in the absence of physical paralysis^66^ Notably, the ACC Cluster network also aligns with an apathy/motivation network identified from behavioural variant frontotemporal dementia (bvFTD).^67^ Moreover, the ACC Cluster spans both canonical large-scale executive control networks, i.e. fronto-parietal and cingulo-opercular.^68^ We observe overlap with regions of the salience network, characterized by anterior insula/opercular–ACC connectivity, which guides motivational drive by identifying emotionally or behaviourally significant stimuli and allocating attention accordingly.^69^ The ACC Cluster and salience networks share connectivity with the ACC and basal ganglia, regions central to motivation and initiation of goal-directed behaviour.^58,59^ Supporting this motivation-based characterization, the ACC Cluster also includes connectivity to a key node in the dlPFC, a central region of the fronto-parietal control network, which is involved in moment-to-moment decision-making in goal-directed behaviour.^70^ Previous work has behaviourally linked motivation and aggression, suggesting that individuals with increased approach motivation are more likely to show aggressive inclinations.^71,72^ Overall, the convergence of these networks may indicate a behavioural phenotype of aggression involving a disruption in the modulation of motivation.

Our second cluster is characterized by connectivity to the vmPFC, OFC, anterior temporal lobes, hippocampus and amygdala. The amygdala and hippocampus seem to be of particular importance in impulsive aggression^73-77^. Specifically, the amygdala appears to instantiate aggressive behaviour via disruptions to emotional reactivity,^55,75-77^ while the hippocampus may be related to aggression via disruptions in social learning.^78^ Similarly, the anterior temporal lobes play a key role in social and emotional information processing. Interestingly, while injuries to this region can lead to increased anger and hostility, they do not typically result in aggressive behaviour unless other areas of the brain are also affected.^28^ Consistent with the outlined characterization of aggression, this cluster not only demonstrates a disruption of these emotion-related areas but also of cognitive control areas, particularly regions of the frontal lobe which are specifically linked to emotion modulation.^79^ This vmPFC Cluster network was similar to the disinhibition network identified from an atrophy network mapping of bvFTD,^67^ sharing similar positive connections to the vmPFC, lateral OFC and anterior temporal lobes. Overall, these connections seem to support the possibility of aggression resulting from impaired inhibition.

Consistent with criminal behaviour models emphasizing impulsivity and deficient self-control,^79,80^ both our vmPFC Cluster network and the described bvFTD disinhibition networks were highly similar to a previously identified ‘criminal behaviour’ network, further supporting a disinhibition-based alteration of cognitive processing.^42^ Given that deficient self-control is a central construct in dominant theories of criminality, these findings may indicate that the disinhibition observed here reflects a broader impairment in vmPFC-mediated self-regulatory processes. The vmPFC Cluster also has a higher rate of criminal behaviour compared with the ACC Cluster (∼17% higher). Studies of offenders consistently find differences in the regions identified in the vmPFC Cluster, specifically the frontal and temporal regions,^80,81^ and have found changes to these regions to be predictive of recidivism.^82^ Additionally, the vmPFC Cluster overlaps with key nodes of the default mode network (DMN), most notably the vmPFC itself.^83,84^ The DMN is active during prospective thinking and inferring other’s intentions, while also mediating cognitive empathy.^70^ Disruption of this network may impair the ability to anticipate consequences or understand others’ mental states, which could contribute to not only aggression but also a predisposition to criminal behaviour, especially when combined with disinhibition.

While all patients in the vmPFC Cluster shared similar positive connections to medial frontal and anterior temporal regions, only the subset of patients with reported crime had significant negative connections to the occipital lobe and significant positive connections to the middle temporal gyrus. Previous work has shown that, compared with healthy males, violent offenders had increased white matter volume in the occipital lobe.^75^ Additionally, disruptions to the occipital and temporal regions may impact behaviour via alterations to the processing of provocative stimuli.^19^ Overall, this suggests that certain connections may be more related to crime or an increased vulnerability to committing criminal acts.

Overall, the distinct ACC and vmPFC aggression networks, and their association with different syndromes and behavioural traits, suggest that there may be unique behavioural features across the patients in our cohort. Identifying and understanding these network differences may be critically important for designing new treatment modalities for aggression. Two key factors in non-invasive neuromodulation-based therapy are identifying the optimal location for applied neuromodulation and the direction of stimulation (i.e. increasing or decreasing bulk brain activity in a targeted region). Notably, the clusters have different, often non-overlapping, key nodes. Unfortunately, case reports are heterogeneous in behavioural reporting; salient details such as the presence of endogenous or exogenous stimuli, the presence of remorse or other details may be lacking. In addition, the conceptualization of aggression itself is broad within the scientific literature and may encompass very different processes. Future research evaluating different behavioural phenotypes in a prospective manner may identify distinctive features of these networks, helping to establish behavioural correlates and better individualize treatment targets.

### Limitations and future directions

While this study represents the largest collection of case reports of lesions strongly related to aggression to date, there are still several limitations to what we can conclude. First, although our cohort includes 61 subjects—the largest number assembled to date—it remains a relatively small sample size and is compiled from many independent clinical observations with inconsistent characterization, including variability in both pre- and post-injury behavioural descriptions. Additionally, the age at which the injury occurred varied widely across participants, which introduces additional variability. As such, the findings should be interpreted with appropriate caution. However, such variability would likely bias us against the consistent results found here.

Second, our search strategy did not include the terms ‘injury’ or ‘injuries’ as our aim was to identify focal brain lesions with imaging suitable for lesion network mapping rather than broader categories of brain injury (e.g. diffuse traumatic injury) that typically lack discrete lesion localization, though this approach may have led to omission of some reports that used broader injury terminology for focal damage. Third, the accuracy of our lesion tracings is limited by the quality of the images published and the fact that we are only able to create 2D representations of each lesion. However, prior work has demonstrated that leveraging these 2D renderings can produce very similar results to those based on full 3D segmentations.^35,38,85^ In addition, although inter-rater reliability was not formally assessed, all tracings were reviewed by a neurologist with neuroimaging expertise (A.L.C.), and lesion network mapping stands as a relatively robust technique with respect to minor tracing differences due to the reliance on low-dimensional connectivity patterns derived from large normative datasets with inherent variance.^35,86^ Another aspect to highlight is that we used a normative connectome to approximate lesion connectivity since it was not possible to obtain pre-lesion functional imaging from the patients themselves. As such, there is likely some level of individual variation in whether specific connections to the predicted regions are actually disrupted in each individual patient in the cohort. Nonetheless, work with individual patient post-stroke functional connectivity data versus normative connectome data has demonstrated that the ability to localize these converging networks is still feasible and effective with normative data.^87^

Prospective acquisition of behavioural data characterizing aggression before and after brain injury may clarify the functional differences between the two networks identified here and would allow a more fine-grained characterization of the aggressive behaviour, as well as the behavioural phenotype overall. Additionally, this would allow us to obtain a more complete history to better characterize pre-lesion behaviour, such as whether patients exhibited aggressive tendencies or criminal behaviour prior to the injury. Furthermore, previous work suggests that some of the key regions identified in this study may be sexually dimorphic, wherein opposite directions of activity have a convergent behavioural effect.^88^ Given the relationship of sex and aggression, prospective work will help elucidate how disruptions to the identified networks may cause variable behavioural changes between sex/gender. We also suspect that neuromodulation to nodes of these networks will help provide further insight into the practical implications of injury or alteration of the networks localized here.

## Conclusion

Here, we compiled the largest case series of lesion-induced aggression and related behaviours to date and applied lesion network mapping to identify neural circuitry that is commonly affected in these 61 patients. We identified two distinct clusters of lesions based on their connectivity patterns, suggesting heterogeneity in the neural pathways leading to aggression. Our analysis also differentiated between lesion connectivity associated with aggressive behaviours and those linked to criminal behaviours, indicating overlapping yet distinct neural underpinnings. Additionally, our study suggests that stratifying patients based on alterations of their connectivity patterns may enhance both diagnosis of aggression and the effectiveness of neuromodulation therapies.

## Supplementary Material

fcag032_Supplementary_Data

## Data Availability

The functional connectivity data equivalent to that used in this study is available online through the Harvard Dataverse at: https://doi.org/10.7910/DVN/ILXIKS and the pipeline used to prepare the functional connectivity data is available at: https://github.com/nimlab. Aggression Case Lesion Tracings are available upon reasonable request. Boston Lesion Repository data requests should be directed to the Center for Brain Circuit Therapeutics at: https://github.com/nimlab.
