# Supplementary material for: Lesion network mapping of focal injury-related aggression finds two distinct network injury patterns
Source: Brain Commun. 2026 Feb 2;8(1):fcag032. doi: 10.1093/braincomms/fcag032 (PMC12914467; doi:10.1093/braincomms/fcag032)

**Supplementary Table 1: Cases of Acquired Aggression.**

| Case # | Reference | Etiology | Age of Injury | Sex |
| --- | --- | --- | --- | --- |
| 1 | Alkawadri 2011 case 1 [1] | Trauma | 38 y/o | M |
| 2 | Anderson 1999 case 1* [2] | Trauma | 15 months | F |
| 3 | Anderson 1999 case 2* [2] | Tumor | 3 months | M |
| 4 | Balcioglu 2020 [3] | Stroke | 34 y/o | M |
| 5 | Benabarre 2001 [4] | Surgical Injury | 20 y/o | M |
| 6 | Blair 2000* [5] | Trauma | 56 y/o | F |
| 7 | Boes 2011* [6] | Congenital Malformation | Congenital | M |
| 8 | Choi 2018 [7] | Unknown | 43 y/o | F |
| 9 | Daglioglu 2008 [8] | Tumor | 21 y/o | M |
| 10 | Daum & Ackerman 1994 [9] | Stroke | 64 y/o | M |
| 11 | de Almeida 2008 [10] | Hamartoma | Congenital | M |
| 12 | DeLong 2002 case 1 [11] | Trauma | Mid-fetal | M |
| 13 | DeLong 2002 case 2 [11] | Trauma | 5 y/o | F |
| 14 | Diana 2021 [12] | Tumor | 39 y/o | M |
| 15 | Genis 2019 [13] | Cyst | 40 y/o | F |
| 16 | Gipson 2017 [14] | Tubers (TSC) | 5 y/o | M |
| 17 | Goh 2015 [15] | Stroke | 33 y/o | F |
| 18 | Hennessy 2001 [16] | Tumor | 9 y/o | M |
| 19 | Hu 2021 [17] | Encephalitis | 23 y/o | F |
| 20 | Maiellaro 2021 [18] | Tumor | 20 y/o | M |
| 21 | Markoula 2012 [19] | Encephalitis | 44 y/o | F |
| 22 | Martin 1992 case 1 [20] | Granuloma | 30 y/o | M |
| 23 | Martinius 1983* [21] | Unknown | 14 y/o ** | M |
| 24 | Michali 2021 [22] | Abscess | 39 y/o | M |
| 25 | Mitchell 2006 case BM* [23] | Tumor | 32 y/o | M |
| 26 | Mitchell 2006 case CL*[23] | Trauma | 26 y/o | M |
| 27 | Mitchell 2006 case DK* [23] | Trauma | 14 y/o | M |
| 28 | Muller 2011* [24] | Unknown | 16 y/o | M |
| 29 | Nakaji 2003 case 1* [25] | Tumor | 5 y/o | M |
| 30 | Nakaji 2003 case 2* [25] | Tumor | 4 y/o | M |
| 31 | Nombora 2022 [26] | Tumor | 54 y/o | M |
| 32 | Novak 2007 [27] | Lipoma | 58 y/o | M |
| 33 | Orellana 2013* [28] | Surgical Injury | 40 y/o | F |
| 34 | Rangwala 2017 [29] | Tumor | 8 y/o | M |
| 35 | Sansing 2007 [30] | Encephalitis | 34 y/o | F |
| 36 | Schiltz 2013 case a* [31] | Unknown | 40 y/o ** | M |
| 37 | Schiltz 2013 case c* [31] | Unknown | 61 y/o ** | M |
| 38 | Schiltz 2013 case d* [31] | Unknown | 49 y/o ** | M |
| 39 | Schiltz 2013 case e* [31] | Unknown | 52 y/o ** | M |
| 40 | Schiltz 2013 case h* [31] | Unknown | 31 y/o ** | M |
| 41 | Schiltz 2013 case l* [31] | Unknown | 45 y/o ** | M |
| 42 | Sener 2015* [32] | Trauma | 7 y/o | M |
| 43 | Spengos 2005 [33] | Stroke | 67 y/o | M |
| 44 | Srichawla 2022 [34] | Encephalitis | 59 y/o | M |
| 45 | Suzuki 1992 [35] | Stroke | 63 y/o | F |
| 46 | Tonkonogy 1991 case 1* [36] | Abscess resection | 16 y/o | F |
| 47 | Tonkonogy 1991 case 2* [36] | Trauma | 23 y/o | M |
| 48 | Tonkonogy 1991 case 3* [36] | Unknown | 21 y/o | M |
| 49 | Tonkonogy 1992 case 1 [37] | Tumor | 18 y/o | M |
| 50 | Trebuchon 2013 case 1* [38] | Cortical dysplasia | 16 y/o | F |
| 51 | Vences 2022 [39] | Encephalitis | 72 y/o | M |
| 52 | Villano 2009* [40] | Tumor | 55 y/o | M |
| 53 | Witzel 2016 case a* [41] | Unknown | 25 y/o ** | M |
| 54 | Witzel 2016 case c* [41] | Unknown | 77 y/o ** | M |
| 55 | Witzel 2016 case d* [41] | Unknown | 57 y/o ** | M |
| 56 | Witzel 2016 case g* [41] | Unknown | 38 y/o ** | M |
| 57 | Witzel 2016 case h* [41] | Unknown | 43 y/o ** | M |
| 58 | Xu 2007 [42] | Tumor | 23 y/o | F |
| 59 | Yadav 2010 [43] | Multiple Sclerosis | 18 y/o | M |
| 60 | Yang 2010 [44] | Prion Disease | 34 y/o | M |
| 61 | Yang 2015 [45] | Encephalitis | 37 y/o | M |

** = Patient included in Darby et al., 2018 cohort; **= Age at Scan given since Age at Injury unknown*

References

[1] R. Alkawadri, B. E. Mickey, C. J. Madden, and P. C. Van Ness, “Cingulate Gyrus Epilepsy: Clinical and Behavioral Aspects, With Surgical Outcomes,” *Archives of Neurology*, vol. 68, no. 3, pp. 381–385, Mar. 2011, doi: 10.1001/archneurol.2011.21.

[2] S. W. Anderson, A. Bechara, H. Damasio, D. Tranel, and A. R. Damasio, “Impairment of social and moral behavior related to early damage in human prefrontal cortex,” *nature neuroscience*, vol. 2, no. 11, 1999.

[3] Y. H. Balcioglu, M. Dogan, I. Inci, and M. Solmaz, “Sexual Behavioral Disinhibition Associated with Nucleus Lentiformis Lesion: A Forensic Neuroscience Perspective Through a Case,” *J Forensic Sci*, vol. 65, no. 5, pp. 1779–1783, Sep. 2020, doi: 10.1111/1556-4029.14477.

[4] A. Benabarre, “Neuropsychological and psychiatric complications in endoscopic third ventriculostomy: a clinical case report,” *Journal of Neurology, Neurosurgery & Psychiatry*, vol. 71, no. 2, pp. 268–271, Aug. 2001, doi: 10.1136/jnnp.71.2.268.

[5] R. J. R. Blair, “Impaired social response reversal: A case of `acquired sociopathy’,” *Brain*, vol. 123, no. 6, pp. 1122–1141, Jun. 2000, doi: 10.1093/brain/123.6.1122.

[6] A. D. Boes, A. H. Grafft, C. Joshi, N. A. Chuang, P. Nopoulos, and S. W. Anderson, “Behavioral effects of congenital ventromedial prefrontal cortex malformation,” *BMC Neurol*, vol. 11, p. 151, Dec. 2011, doi: 10.1186/1471-2377-11-151.

[7] B. S. Choi, G. Shen, G. Nan, J.-M. Kim, K.-Y. Jung, and B. Jeon, “Dramatic psychiatric and behavioral symptoms following a subthalamic lesion,” *J Clin Neurosci*, vol. 47, pp. 154–156, Jan. 2018, doi: 10.1016/j.jocn.2017.10.051.

[8] E. Daglioglu, O. Okay, A. Dalgic, A. L. Albayrak, and F. Ergungor, “Cystic olfactory schwannoma of the anterior cranial base,” *Br J Neurosurg*, vol. 22, no. 5, pp. 697–699, Oct. 2008, doi: 10.1080/02688690801983654.

[9] I. Daum and H. Ackermann, “Frontal-type memory impairment associated with thalamic damage,” *Int J Neurosci*, vol. 77, no. 3–4, pp. 187–98, Aug. 1994, doi: 10.3109/00207459408986030.

[10] A. N. de Almeida, E. T. Fonoff, G. Ballester, M. J. Teixeira, and R. Marino, “Stereotactic disconnection of hypothalamic hamartoma to control seizure and behavior disturbance: case report and literature review,” *Neurosurgical review*, vol. 31, no. 3, pp. 343–348, 2008, doi: 10.1007/s10143-008-0142-8.

[11] G. R. DeLong, “Mid-gestation right basal ganglia lesion - Clinical observations in two children,” *Neurology*, vol. 59, no. 1, pp. 54–58, 2002, doi: 10.1212/WNL.59.1.54.

[12] L. L. Diana, J. Carmona-Huerta, J. G. Patino, A. L. Alejandro, and D. A. Sol, “Atypical Charles Bonnet syndrome secondary to frontal meningioma: a case report,” *BMC Psychiatry*, vol. 21, no. 1, p. 365, Jul. 2021, doi: 10.1186/s12888-021-03360-6.

[13] B. Genis and B. Cosar, “A Case of Arachnoid Cyst Presenting with Cognitive Impairment and Hypomania Symptoms,” *Turkish Journal of Psychiatry*, 2019, doi: 10.5080/u23937.

[14] T. T. Gipson and A. Poretti, “Implementing a Multidisciplinary Approach to Treating Tuberous Sclerosis Complex: A Case Report.,” *Child Neurol Open*, vol. 4, p. 2329048X17725609, Dec. 2017, doi: 10.1177/2329048X17725609.

[15] K. G. Goh and V. Shanthi, “The Importance of Early Recognition of Cerebral Venous Sinus Thrombosis: A Case Report,” *Malays J Med Sci*, vol. 22, no. 5, pp. 98–102, Sep. 2015.

[16] M. J. Hennessy, M. Koutroumanidis, E. Hughes, and C. D. Binnie, “Psychomotor EEG variant of Gibbs: an association with underlying structural pathology,” *Clinical Neurophysiology*, vol. 112, no. 4, pp. 686–687, Apr. 2001, doi: 10.1016/S1388-2457(01)00472-2.

[17] S. Hu, T. Lan, R. Bai, S. Jiang, J. Cai, and L. Ren, “HSV encephalitis triggered anti-NMDAR encephalitis: a case report,” *Neurol Sci*, vol. 42, no. 3, pp. 857–861, Mar. 2021, doi: 10.1007/s10072-020-04785-9.

[18] A. Maiellaro, A. Perna, P. Giugliano, M. Esposito, and G. Vacchiano, “Sudden Death from Primary Cerebral Melanoma: Clinical Signs and Pathological Observations,” *Healthcare (Basel)*, vol. 9, no. 3, p. 341, Mar. 2021, doi: 10.3390/healthcare9030341.

[19] S. Markoula, D. Chatzistefanidis, S. Konitsiotis, and A. P. Kyritsis, “An emerging problem in clinical practice: how to approach acute psychosis,” *Clin Pract*, vol. 2, no. 1, p. e7, Jan. 2012, doi: 10.4081/cp.2012.e7.

[20] J. B. Martin and P. N. Riskind, “Neurologic manifestations of hypothalamic disease,” *Prog Brain Res*, vol. 93, pp. 31–40; discussion 40-42, 1992, doi: 10.1016/s0079-6123(08)64561-6.

[21] Martinius, “Homicide of an aggressive adolescent boy with right temporal lesion: A case report,” *Neuroscience & Biobehavioral Reviews*, vol. 7, no. 3, pp. 419–422, Sep. 1983, doi: 10.1016/0149-7634(83)90048-9.

[22] M. C. Michali, I. G. Kastanioudakis, L. V. Basiari, G. Alexiou, and I. D. Komnos, “Parenchymal Brain Abscess as an Intracranial Complication After Sinusitis,” *Cureus*, vol. 13, no. 8, p. e17365, Aug. 2021, doi: 10.7759/cureus.17365.

[23] D. G. V. Mitchell *et al.*, “Instrumental learning and relearning in individuals with psychopathy and in patients with lesions involving the amygdala or orbitofrontal cortex.,” *Neuropsychology*, vol. 20, no. 3, pp. 280–289, May 2006, doi: 10.1037/0894-4105.20.3.280.

[24] J. L. Müller, “Are Sadomasochism and Hypersexuality in Autism Linked to Amygdalohippocampal Lesion?,” *The Journal of Sexual Medicine*, vol. 8, no. 11, pp. 3241–3249, Nov. 2011, doi: 10.1111/j.1743-6109.2009.01485.x.

[25] P. Nakaji, H. S. Meltzer, S. A. Singel, and J. F. Alksne, “Improvement of aggressive and antisocial behavior after resection of temporal lobe tumors,” *Pediatrics*, vol. 112, no. 5, p. e430, Nov. 2003, doi: 10.1542/peds.112.5.e430.

[26] O. Nombora, A. Miguel, L. Lopes, and Â. Venâncio, “The Intriguing Diagnosis of Lung Cancer in a Psychiatry Inpatient Unit: A Reflection Through a Case Report,” *Cureus*, vol. 14, no. 9, p. e29450, Sep. 2022, doi: 10.7759/cureus.29450.

[27] Z. Novak, J. Chrastina, and E. Lzicarova, “Exceptional symptomatology of a lipoma beneath the third ventricular floor and successful neuroendoscopic treatment,” *Minim Invasive Neurosurg*, vol. 50, no. 1, pp. 56–59, Feb. 2007, doi: 10.1055/s-2007-970058.

[28] G. Orellana, L. Alvarado, C. Muñoz-Neira, R. Ávila, M. F. Méndez, and A. Slachevsky, “Psychosis-related matricide associated with a lesion of the ventromedial prefrontal cortex,” *J Am Acad Psychiatry Law*, vol. 41, no. 3, pp. 401–406, 2013.

[29] S. D. Rangwala, M. K. Tobin, D. M. Birk, J. T. Butts, D. C. Nikas, and Y. S. Hahn, “Pica in a Child with Anterior Cingulate Gyrus Oligodendroglioma: Case Report,” *Pediatr Neurosurg*, vol. 52, no. 4, pp. 279–283, 2017, doi: 10.1159/000477816.

[30] L. H. Sansing, E. Tuzun, M. W. Ko, J. Baccon, D. R. Lynch, and J. Dalmau, “A patient with encephalitis associated with NMDA receptor antibodies,” *Nat Clin Pract Neurol*, vol. 3, no. 5, pp. 291–6, May 2007, doi: 10.1038/ncpneuro0493.

[31] K. Schiltz, J. Witzel, J. Bausch-Hölterhoff, and B. Bogerts, “High prevalence of brain pathology in violent prisoners: a qualitative CT and MRI scan study,” *European Archives of Psychiatry & Clinical Neuroscience*, vol. 263, no. 7, pp. 607–616, Oct. 2013, doi: 10.1007/s00406-013-0403-6.

[32] M. T. Sener, H. Ozcan, S. Sahingoz, and H. Ogul4, “Criminal Responsibility of the Frontal Lobe Syndrome,” *Eurasian J Med*, vol. 47, no. 3, pp. 218–222, Oct. 2015, doi: 10.5152/eurasianjmed.2015.69.

[33] K. Spengos, J. C. Wohrle, G. Tsivgoulis, G. Stouraitis, K. Vemmos, and V. Zis, “Bilateral paramedian midbrain infarct: an uncommon variant of the ‘top of the basilar’ syndrome,” *J Neurol Neurosurg Psychiatry*, vol. 76, no. 5, pp. 742–743, May 2005, doi: 10.1136/jnnp.2004.050146.

[34] B. S. Srichawla, “Autoimmune Voltage-Gated Potassium Channel Limbic Encephalitis With Auditory and Visual Hallucinations,” *Cureus*, vol. 14, no. 5, p. e25186, May 2022, doi: 10.7759/cureus.25186.

[35] T. Suzuki *et al.*, “Changes in personality and emotion following bilateral infarction of the posterior cerebral arteries,” *Jpn J Psychiatry Neurol*, vol. 46, no. 4, pp. 897–903, Dec. 1992, doi: 10.1111/j.1440-1819.1992.tb02858.x.

[36] J. M. Tonkonogy, “Violence and temporal lobe lesion: head CT and MRI data,” *JNP*, vol. 3, no. 2, pp. 189–196, May 1991, doi: 10.1176/jnp.3.2.189.

[37] J. M. Tonkonogy and J. L. Geller, “Hypothalamic lesions and intermittent explosive disorder,” *J Neuropsychiatry Clin Neurosci*, vol. 4, no. 1, pp. 45–50, 1992, doi: 10.1176/jnp.4.1.45.

[38] A. Trebuchon, F. Bartolomei, A. McGonigal, V. Laguitton, and P. Chauvel, “Reversible antisocial behavior in ventromedial prefrontal lobe epilepsy,” *Epilepsy & Behavior*, vol. 29, no. 2, pp. 367–373, Nov. 2013, doi: 10.1016/j.yebeh.2013.08.007.

[39] M. A. Vences *et al.*, “Post-Vaccinal Encephalitis with Early Relapse after BNT162b2 (COMIRNATY) COVID-19 Vaccine: A Case Report.,” *Vaccines (Basel)*, vol. 10, no. 7, Jul. 2022, doi: 10.3390/vaccines10071065.

[40] J. L. Villano, N. Mlinarevich, K. S. Watson, H. H. Engelhard, and L. Anderson-Shaw, “Aggression in a patient with primary brain tumor: ethical implications for best management,” *J Neurooncol*, vol. 94, no. 2, pp. 293–296, Sep. 2009, doi: 10.1007/s11060-009-9850-3.

[41] J. G. Witzel, B. Bogerts, and K. Schiltz, “Increased frequency of brain pathology in inmates of a high-security forensic institution: a qualitative CT and MRI scan study,” *Eur Arch Psychiatry Clin Neurosci*, vol. 266, no. 6, pp. 533–541, Sep. 2016, doi: 10.1007/s00406-015-0620-2.

[42] J. Xu *et al.*, “A growth in bipolar disorder?,” *Acta Psychiatr Scand*, vol. 115, no. 3, pp. 246–50; discussion 250, Mar. 2007, doi: 10.1111/j.1600-0447.2006.00907.x.

[43] R. Yadav and A. S. Zigmond, “Temporal lobe lesions and psychosis in multiple sclerosis,” *BMJ Case Rep*, vol. 2010, p. bcr0120102651, Oct. 2010, doi: 10.1136/bcr.01.2010.2651.

[44] C. W. Yang, J. L. Fuh, S. J. Wang, J. F. Lirng, C. C. Yang, and S. J. Cheng, “Probable variant Creutzfeldt–Jakob disease in Asia: a case report from Taiwan and review of two prior cases,” *Psychiatry Clin Neurosci*, vol. 64, no. 6, pp. 652–8, Dec. 2010, doi: 10.1111/j.1440-1819.2010.02151.x.

[45] Y. Yang, J. Xiao, H. Song, R. Wang, M. Hussain, and W. Song, “Relationship of herpes simplex encephalitis and transcranial direct current stimulation--a case report,” *J Clin Virol*, vol. 65, pp. 46–9, Apr. 2015, doi: 10.1016/j.jcv.2015.01.012.

**Supplementary Figure 1: Lesions Associated with Aggression.** Representative slices of the 61 lesions associated with aggressive behavior.


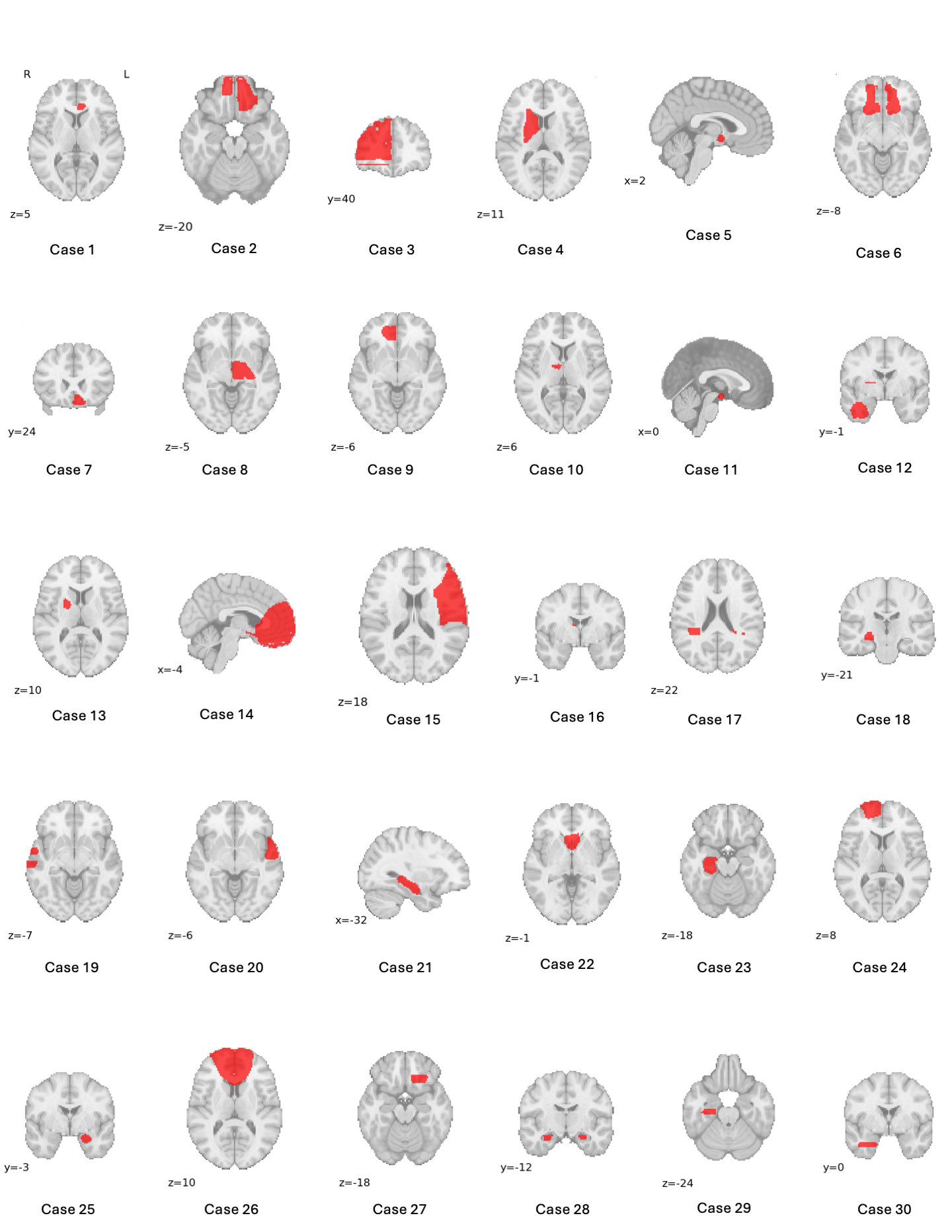


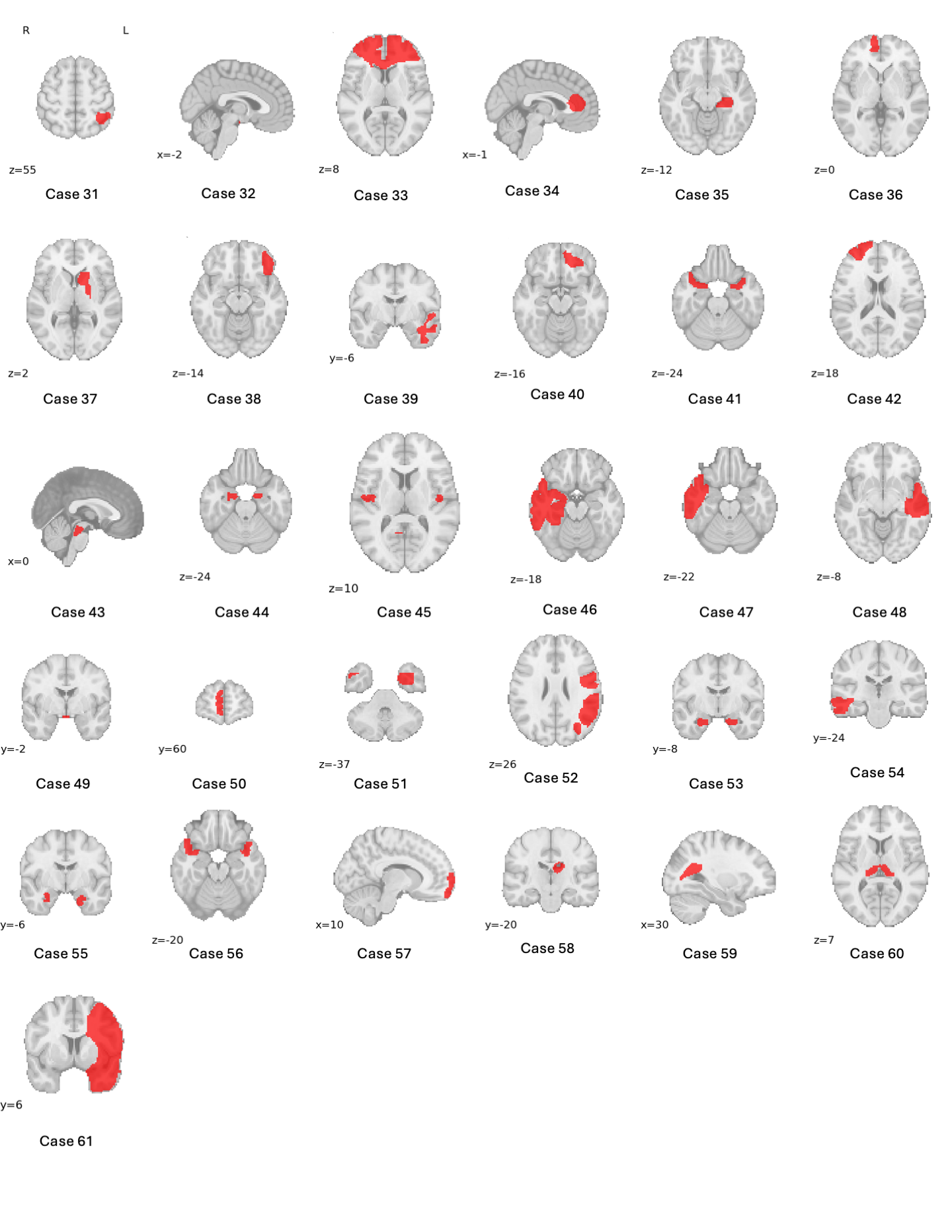


**Supplementary Table 2: Control Lesions from the Boston Lesion Repository**

| **Symptom** | **Number subjects** | **Citation** | **Doi** |
| --- | --- | --- | --- |
| Akinetic mutism | 28 | Darby et al., 2018 [1] | 10.1073/pnas.1814117115 |
| Alien limb | 53 | Darby et al., 2018 [1] | 10.1073/pnas.1814117115 |
| Amnesia | 53 | Ferguson et al., 2019 [2] | 10.1038/s41467-019-11353-z |
| Anton syndrome | 23 | Kletenik et al. 2023 [3] | 10.1002/ana.26709 |
| Aphasia | 12 | Boes et al., 2015 [4] | 10.1093/brain/awv228 |
| Asterixis | 30 | Joutsa et al., 2018 [5] | 10.1002/ana.25285 |
| Blindsight | 34 | Kletenik et al., 2021 [6] | 10.1002/ana.26292 |
| Coma | 12 | Fischer et al., 2016 [7] | 10.1212/WNL.0000000000003404 |
| Confabulation | 25 | Bateman et al., 2023 [8] | 10.1176/appi.neuropsych.20220160 |
| Cortical blindness | 35 | Kletenik et al., 2021 [6] | 10.1002/ana.26292 |
| Delusion (Capgras & Other) | 32 | Darby et al., 2017 [9] | 10.1093/brain/aww288 |
| Cervical dystonia | 25 | Corp et al., 2019 [10] | 10.1093/brain/awz112 |
| Freezing of gait | 14 | Fasano et al., 2017 [11] | 10.1002/ana.24845 |
| Hallucination | 89 | Boes et al. 2015 Brain, [4]  Kim et al. 2021 Mol Psych [12] | 10.1093/brain/awv228,  10.1038/s41380-019-0565-3 |
| Hemichorea | 29 | Laganiere et al., 2016 [13] | 10.1212/WNL.0000000000002741 |
| Holmes tremor | 36 | Joutsa et al., 2019 [14] | 10.1002/ana.25618 |
| Infantile spasms | 74 | Cohen et al., 2021 [15] | 10.1002/ana.26015 |
| Loss of consciousness | 16 | Snider et al., 2020 [16] | 10.1002/hbm.24892 |
| Central poststroke pain | 23 | Boes et al., 2015 [4] | 10.1093/brain/awv228 |
| Parkinsonism | 29 | Joutsa et al., 2018 [17] | 10.1093/brain/awy161 |
| Prosopagnosia | 44 | Cohen et al., 2019 [18] | 10.1093/brain/awz332 |

References

[1] R. R. Darby, J. Joutsa, M. J. Burke, and M. D. Fox, “Lesion network localization of free will,” *PNAS*, vol. 115, no. 42, pp. 10792–10797, Oct. 2018, doi: 10.1073/pnas.1814117115.

[2] M. A. Ferguson *et al.*, “A human memory circuit derived from brain lesions causing amnesia,” *Nature communications*, vol. 10, no. 1, p. 3497, Aug. 2019, doi: 10.1038/s41467-019-11353-z.

[3] I. Kletenik, K. Gaudet, S. Prasad, A. L. Cohen, and M. D. Fox, “Network Localization of Awareness in Visual and Motor Anosognosia,” *Ann Neurol*, vol. 94, no. 3, pp. 434–441, Sep. 2023, doi: 10.1002/ana.26709.

[4] A. D. Boes *et al.*, “Network localization of neurological symptoms from focal brain lesions,” *Brain*, vol. 138, no. Pt 10, pp. 3061–3075, Oct. 2015, doi: 10.1093/brain/awv228.

[5] J. Joutsa *et al.*, “Identifying therapeutic targets from spontaneous beneficial brain lesions,” *Ann Neurol*, vol. 84, no. 1, pp. 153–157, Jul. 2018, doi: 10.1002/ana.25285.

[6] I. Kletenik *et al.*, “Network Localization of Unconscious Visual Perception in Blindsight,” *Ann Neurol*, Dec. 2021, doi: 10.1002/ana.26292.

[7] D. B. Fischer *et al.*, “A human brain network derived from coma-causing brainstem lesions,” *Neurology*, vol. 87, no. 23, pp. 2427–2434, 2016, doi: 10.1212/WNL.0000000000003404.

[8] J. R. Bateman *et al.*, “Network Localization of Spontaneous Confabulation,” *The journal of neuropsychiatry and clinical neurosciences*, pp. appineuropsych20220160–appineuropsych20220160, 2023, doi: 10.1176/appi.neuropsych.20220160.

[9] R. R. Darby, S. Laganiere, A. Pascual-Leone, S. Prasad, and M. D. Fox, “Finding the imposter: brain connectivity of lesions causing delusional misidentifications,” *Brain : a journal of neurology*, vol. 140, no. 2, pp. 497–507, Feb. 2017, doi: 10.1093/brain/aww288.

[10] D. T. Corp *et al.*, “Network localization of cervical dystonia based on causal brain lesions,” *Brain*, vol. 142, no. 6, pp. 1660–1674, Jun. 2019, doi: 10.1093/brain/awz112.

[11] A. Fasano, S. E. Laganiere, S. Lam, and M. D. Fox, “Lesions causing freezing of gait localize to a cerebellar functional network,” *Ann Neurol*, vol. 81, no. 1, pp. 129–141, Jan. 2017, doi: 10.1002/ana.24845.

[12] N. Y. Kim *et al.*, “Lesions causing hallucinations localize to one common brain network,” *Molecular psychiatry*, vol. 26, no. 4, pp. 1299–1309, Apr. 2021, doi: 10.1038/s41380-019-0565-3.

[13] S. Laganiere, A. D. Boes, and M. D. Fox, “Network localization of hemichorea-hemiballismus,” *Neurology*, vol. 86, no. 23, pp. 2187–2195, Jun. 2016, doi: 10.1212/WNL.0000000000002741.

[14] J. Joutsa, L. C. Shih, and M. D. Fox, “Mapping holmes tremor circuit using the human brain connectome,” *Ann Neurol*, vol. 86, no. 6, pp. 812–820, Dec. 2019, doi: 10.1002/ana.25618.

[15] A. L. Cohen *et al.*, “Tuber locations associated with infantile spasms map to a common brain network,” *Annals of neurology*, vol. 89, no. 4, pp. 726–739, Apr. 2021, doi: 10.1002/ana.26015.

[16] S. B. Snider *et al.*, “Cortical lesions causing loss of consciousness are anticorrelated with the dorsal brainstem,” *Human Brain Mapping*, vol. 41, no. 6, pp. 1520–1531, Apr. 2020, doi: 10.1002/hbm.24892.

[17] J. Joutsa, A. Horn, J. Hsu, and M. D. Fox, “Localizing parkinsonism based on focal brain lesions,” *Brain*, vol. 141, no. 8, pp. 2445–2456, Aug. 2018, doi: 10.1093/brain/awy161.

[18] A. L. Cohen, L. Soussand, S. L. Corrow, O. Martinaud, J. J. S. Barton, and M. D. Fox, “Looking beyond the face area: lesion network mapping of prosopagnosia,” *Brain*, vol. 142, no. 12, pp. 3975–3990, Dec. 2019, doi: 10.1093/brain/awz332.

**Supplementary Figure 2: Lesion network localization of aggression.** Two sample t-test comparing functional connectivity lesions causing aggression (n = 61) and a large cohort of lesions causing other symptoms (n = 716) (FWE corrected p<0.05).


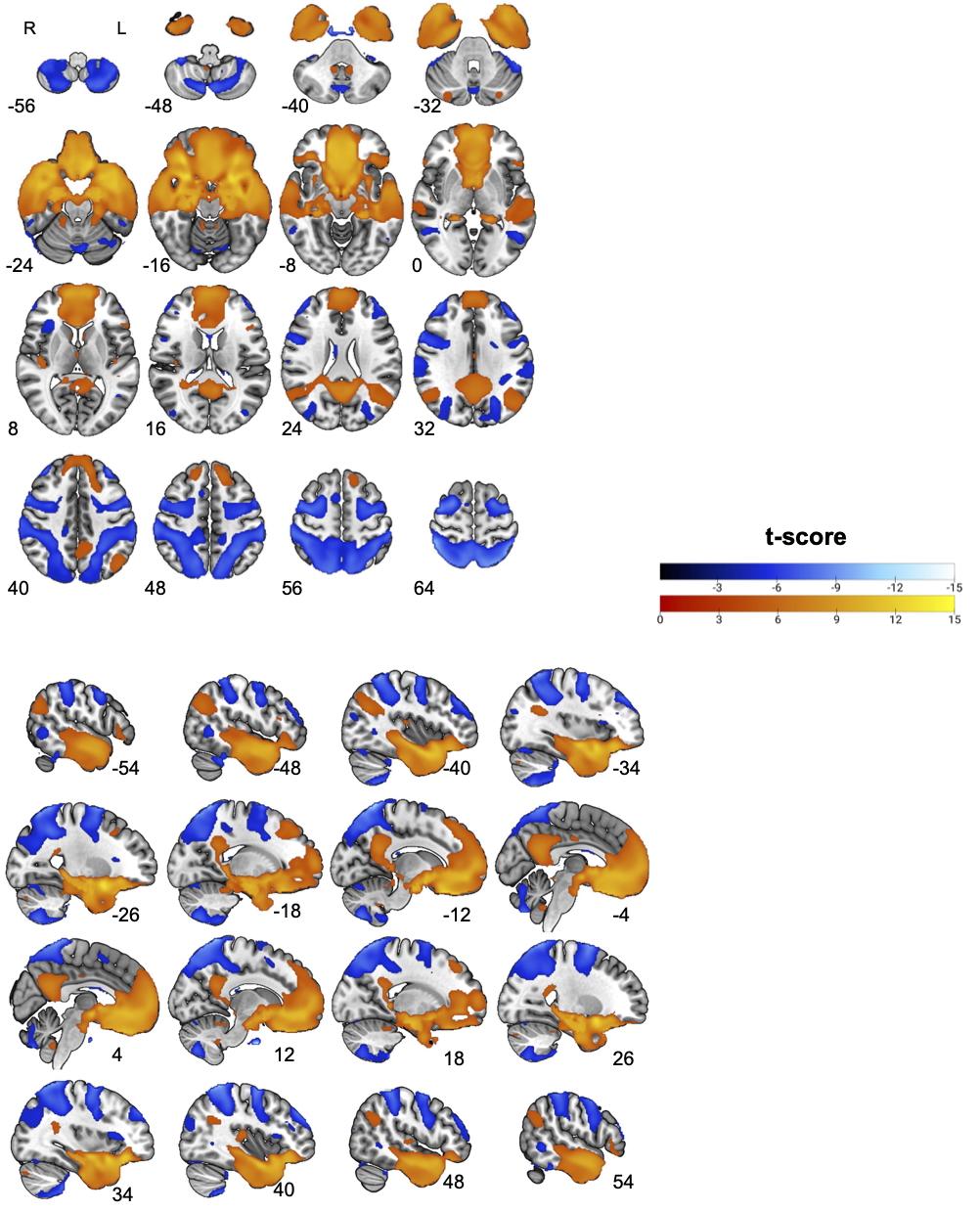


**Supplementary Figure 3: Lesion Connectivity by Sex**. **(A)** Two sample t-test comparing functional connectivity of male (n = 47) vs. female (n = 14) lesion connectivity (uncorrected *P* < 0.05) **(B)** One sample t-test of lesion connectivity of male patients (n=47) (uncorrected *P* < 0.05) **(C)** One sample t-test of lesion connectivity of female patients (n=14) (uncorrected *P* < 0.05). *Top row: z=-18, z=-8, z=-3, x=30; Middle row: z=-18, z=0, z=-14, x=6; Bottom row: z=-24, z=-12, z=12, x=6 (Slices correspond to main figures).*


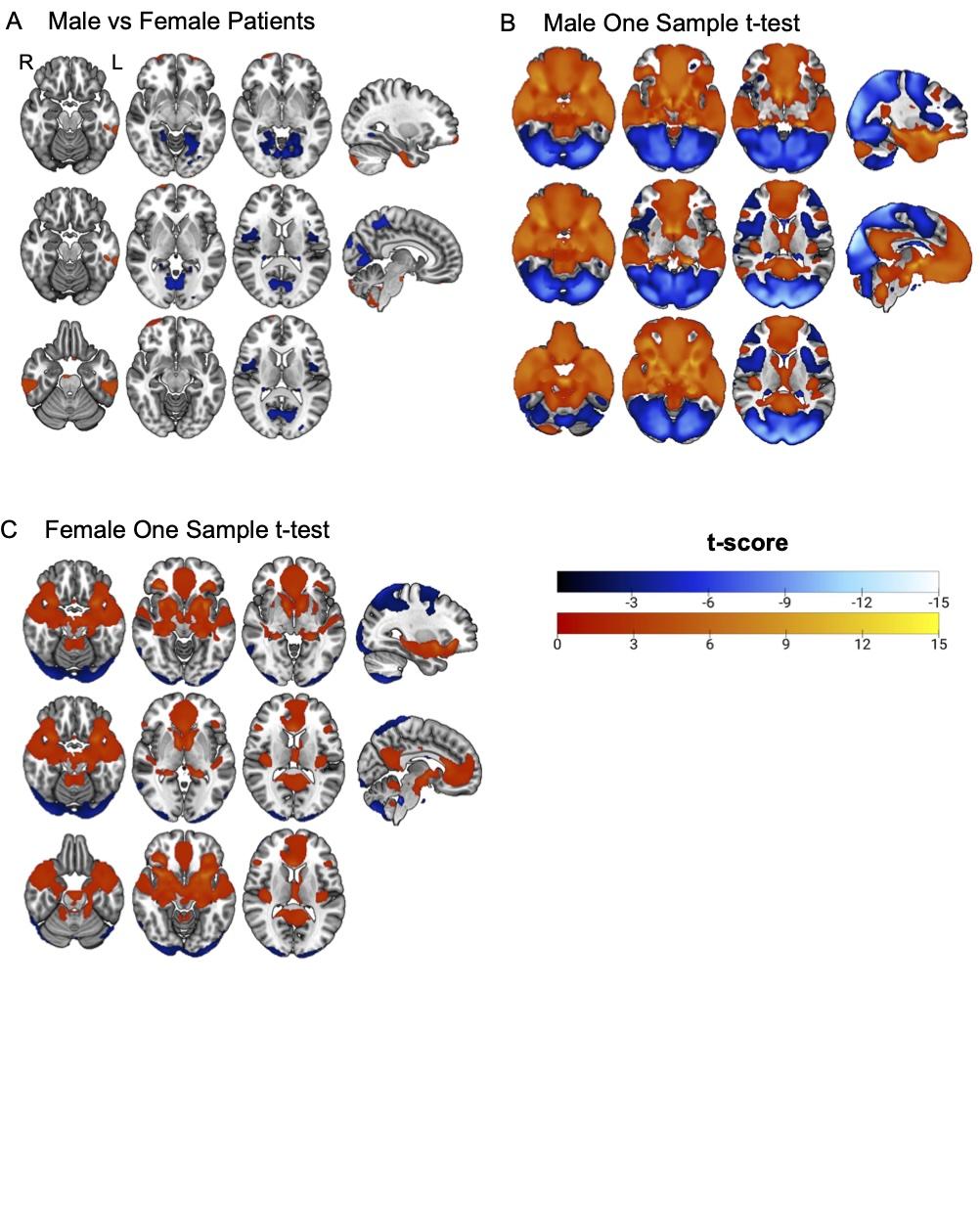

Supplement: fcag032_Supplementary_Data [file fcag032_supplementary_data.docx]
